# Distress and quality of life after autologous stem cell transplantation: a randomized clinical trial to evaluate the outcome of a web-based stepped care intervention

**DOI:** 10.1186/1471-2407-10-361

**Published:** 2010-07-07

**Authors:** Annemarie MJ Braamse, Berno van Meijel, Otto Visser, Patricia van Oppen, Annette D Boenink, Corien Eeltink, Pim Cuijpers, Peter C Huijgens, Aartjan TF Beekman, Joost Dekker

**Affiliations:** 1 Department of Psychiatry and EMGO Institute for Health and Care Research, VU University Medical Center, Amsterdam, the Netherlands; 2 Inholland University, Research Group Mental Health Nursing, Amsterdam, the Netherlands; 3 Department of Hematology, VU University Medical Center, Amsterdam, the Netherlands; 4 Department of Clinical Psychology, FPP, EMGO Institute for Health and Care Research, VU University, Amsterdam, the Netherlands

## Abstract

**Background:**

Psychological distress (i.e. depression and anxiety) is a strong predictor of functional status and other aspects of quality of life in autologous stem cell transplantation following high-dose chemotherapy. Treatment of psychological distress is hypothesized to result in improvement of functional status and other aspects of quality of life. The aim is to evaluate the outcome of stepped care for psychological distress on functional status and other aspects of quality of life in patients with hematological malignancy treated with autologous stem cell transplantation.

**Methods/Design:**

The study is designed as a randomized clinical trial with 2 treatment arms: a stepped care intervention program versus care as usual. Patients are randomized immediately pre transplant. Stepped care and care as usual are initiated after a 6 weeks buffer period. Outcome is evaluated at 13, 30, and 42 weeks post transplant.

In the experimental group, the first step includes an Internet-based self-help program. If psychological distress persists after the self-help intervention, the second step of the program is executed, i.e. a diagnostic evaluation and a standardized interview, yielding a problem analysis. Based on this information, a contract is made with the patient and treatment is provided consisting of individual face-to-face counseling, medication, or referral to other services. Care as usual comprises an interview with the patient, on ad hoc basis; emotional support and advice, on ad hoc basis; if urgent problems emerge, the patient is referred to other services.

Primary outcome variables are psychological distress and functional status. Data are analyzed according to the intention to treat-principle.

**Discussion:**

This study has several innovative characteristics. First, the outcome of the intervention for psychological distress in patients with hematological malignancy treated with autologous stem cell transplantation is evaluated in a randomized controlled study. Second, the impact of the intervention on functional status is evaluated: it is hypothesized that reduction of psychological distress results in improved functional status. Furthermore, the intervention concerns an Internet-based treatment in the first step. Finally, the intervention is characterized by an emphasis on self-management, efficiency, and a multi-disciplinary approach with nurses taking up a central role.

**Trial Registration:**

NTR1770

## Background

### Psychological distress as risk factor for impaired quality of life

Autologous stem cell transplantation (auto-SCT) following high-dose chemotherapy is acknowledged as one of the most stressful treatments in anti-cancer therapy. Previous research documented strong decreases of health-related quality of life during and directly after auto-SCT, with gradual improvement during the first year of follow up [1]. Three to five years after transplantation, most patients report a good health-related quality of life. However, when compared to age- and gender-standardized values of the general population, their health-related quality of life is still decreased [1], in particular with respect to functional status, symptoms, social function, and other aspects of quality of life.

Psychological distress has shown to be the strongest predictor of health-related quality of life apart from relapse in cancer patients following auto-SCT [2-5]. Distress is generally defined as 'a multi-determined unpleasant emotional experience of a psychological (cognitive, behavioral, emotional), social, and/or spiritual nature that may interfere with the ability to cope effectively with cancer, its physical symptoms and its treatment. Distress extends along a continuum, ranging from common feelings of vulnerability, sadness and fears to problems that may become disabling, such as depression, anxiety, panic, social isolation, and spiritual crisis' [6]. Previous research has mainly focused on depression and anxiety as indicators of psychological distress. Approximately 26-36% of patients reports moderate to severe depressive symptoms during the first year post transplant [7,8], 18% of patients endorses moderate to severe anxiety within the first 100 days after transplantation [8].

Patients who suffer from depression before stem cell transplantation, are more likely to have impaired functional status post transplant [2]. Furthermore, the presence of depression and anxiety during the acute phase of transplantation predicts functional status, social function and generic quality of life after transplantation [3]. Similar results have been reported in other studies [4,5]. With respect to depression, some previous studies reported that depression predicts survival after auto-SCT [9-11]. However, the influence of depression on survival in cancer patients is under considerable debate, as no univocal evidence supports this relationship [12].

From the existing literature we may conclude that psychological distress, specifically depression and anxiety, are predictors of functional status and other aspects of quality of life in patients treated with auto-SCT.

### Treatment of psychological distress

The findings on psychological distress being a prognostic determinant of health-related quality of life provide a strong empirical basis for an intervention focusing on treatment of psychological distress in auto-SCT. Treating psychological distress is expected to substantially improve functional status and other aspects of health-related quality of life following auto-SCT.

Problem solving treatment is an effective intervention for reducing psychological distress and improving quality of life in cancer survivors [13-15]. Problem solving treatment does not aim to directly solve patients' problems; instead, it aims to strengthen the self-management skills of patients to solve present and future problems. Patients learn to regain control of their problems and life by (a) prioritizing problems which matter most to them and which in principle can be solved; (b) analyzing the problem and generating alternative solutions; (c) selecting methods for solution and implementing them; and (d) evaluating the results and preparing for the future. The improvement of self-management skills helps patients to adjust to the limitations caused by their disease and to improve their quality of life, to manage their own lives and give direction to the care they receive [16]. Patients receiving auto-SCT may therefore highly profit from improved self-management skills.

In delivering treatment for psychological distress, the stepped care approach has been strongly advocated [17-19]. In this approach, patients start with the least intensive treatment that is most likely to work, with more intensive and costly interventions reserved for those insufficiently helped by the initial intervention. Stepped care attempts to maximize the effectiveness and efficiency of decisions about allocation of resources in therapy. Promising results of this approach have been documented [18].

In the present study, treatment for psychological distress, applying the stepped care approach, will be offered to patients receiving auto-SCT for the treatment of a hematological malignancy. Patients will receive auto-SCT for relapsed disease or upfront. All patients will be pretreated with (immuno-)chemotherapy. The transplant related mortality of auto-SCT is low (<5%). Relapsed disease occurs between 5 and 50% of the patients, depending on their disease.

**Figure 1:**
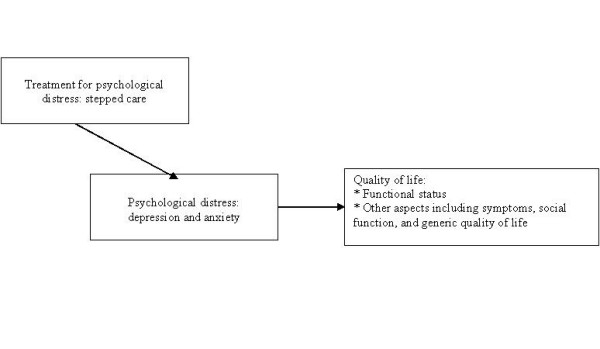
**Stepped care, psychological distress, and quality of life**.

The aim of this study is to evaluate the outcome of stepped care for psychological distress on functional status and other aspects of quality of life in patients with hematological malignancy treated with auto-SCT. It is hypothesized that stepped care results in improvement of psychological distress, and thereby in improvement of functional status and other aspects of quality of life (see figure 1).

## Methods/Design

### Design

**Figure 2:**
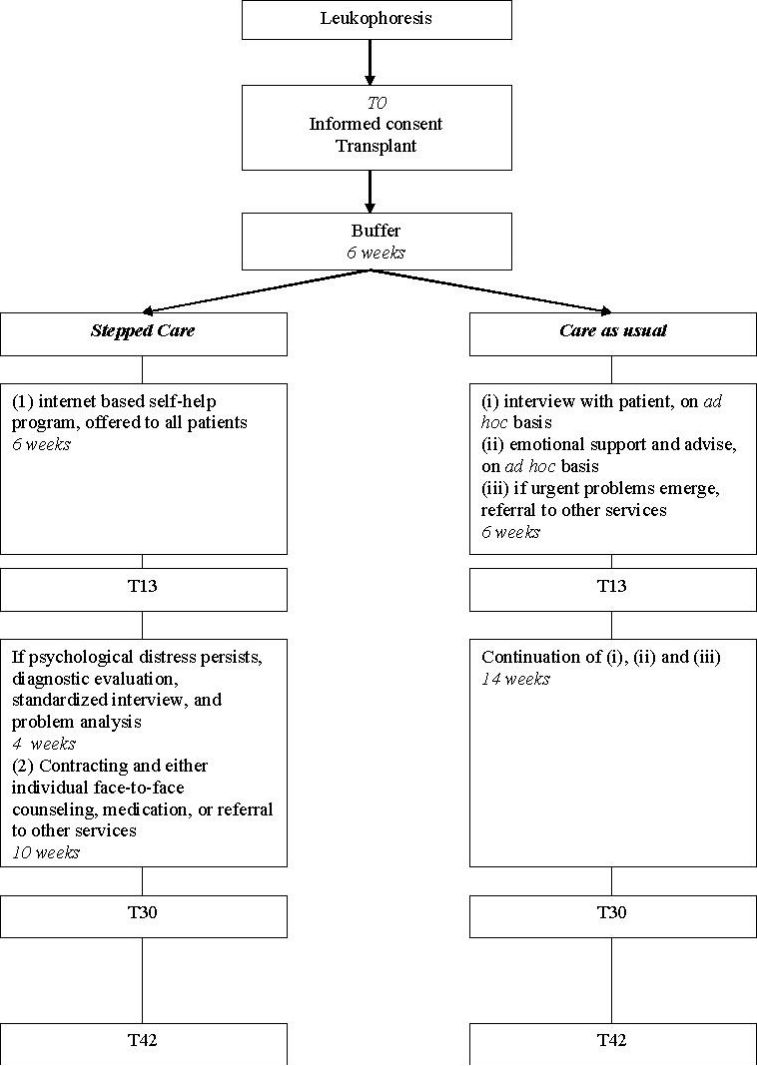
**Design of the RCT**.

The study is designed as a clinical trial with a random allocation of patients into two alternative treatment arms: stepped care and care as usual. Patients treated with auto-SCT are randomized immediately pre-transplant (T0). Stepped care and care as usual are initiated after a 6 weeks buffer period, allowing for initial recovery post-transplant. At 13 weeks (T13), 30 weeks (T30) and 42 weeks (T42) post-transplant, outcome is evaluated. The design is illustrated in figure 2.

### Setting and study sample

This will be a multicenter study in one of the large Dutch university hospitals and a Dutch teaching hospital. *Inclusion criteria*:(a) patients with hematological malignancy (multiple myeloma, (non-)Hodgkin lymphoma, acute myeloid leukemia, or acute lymphoid leukemia) treated with auto-SCT after (immuno-)chemotherapy; and (b) life expectation >3 months. *Exclusion criteria*: (c) age <18 or >65 years (65 years is included); (d) insufficient command of the Dutch language to complete questionnaires; or, if so: no support by family or professional interpreters; (e) contraindication for the stepped care approach; (f) no informed consent.

### Randomization and blinding

To allocate patients to either stepped care or to care as usual, permutated-blocked randomization with stratification for study center (university hospital versus teaching hospital) and for diagnosis (multiple myeloma, (non-)Hodgkin lymphoma, or acute (myeloid or lymphoid) leukemia) made up by a random digit generator is used. When patients have completed the baseline-questionnaire (T0), allocation will be performed by an independent researcher (BM), who is not in contact with the patients and keeps the randomization list in secure and confidential custody. He gives the patient a unique randomization number. The allocation will be sent by email to the investigator (AMJB), who informs the patient. The clinicians involved in the stepped care program will be informed of the allocation by AMJB only after screening for psychological distress at T13, and only if the patient meets the criterion for psychological distress after completion of the Internet based self-help program, resulting in further treatment being offered.

Due to the nature of the intervention, neither patients nor health care providers can be blinded to the intervention. However, randomization, scoring of outcome variables, and statistical analysis will be performed blindly.

### Interventions

#### I. Protocol stepped care

The key-elements of the protocol for stepped care are described below.

#### Step 1: Internet-based self-help program

The Dutch website "Alles onder controle" ["Everything under control"] is used, adjusted for patients with hematological malignancy receiving auto-SCT http://www.allesondercontrole.nu/sct. "Everything under control" is a brief, web based intervention for problem-solving (which is based on self-examination therapy). Both international and national research has shown that this intervention is effective in treating psychological distress [20,21]. The intervention applies the principles of problem-solving therapy, which proved to be effective in several randomized controlled studies [22]. There is also evidence from a growing number of trials showing that psychological treatments can be effectively delivered over the Internet. A recent meta-analysis found that the effects of Internet-based treatments for depression and anxiety disorders are as large as those of face-to-face treatments [23].

The intervention "Everything under control" takes approximately five weeks in total. In that period, respondents describe what they think is important in their lives, make a list of their problems and concerns, and divide these into three categories: important and solvable problems (these are solved through a six-step procedure of problem-solving); unimportant problems (problems that are not related to what is important in their lives); and important but unsolvable problems (such as losing someone through death). For the important and solvable problems, the respondent analyzes the problem and generates alternative solutions; selects and implements the chosen solution; and evaluates the results and prepares for the future. For the important but unsolvable problems, the respondent makes a plan on how to cope with them.

Coaching will be given by one of the researchers (AMJB). The coaching consists of brief, weekly contacts by e-mail, which take about 10 to 15 minutes per week. The total coaching time is 1 to 1,5 hours per respondent. The coaching is not aimed at developing a patient-therapist relationship but is only meant to give support in working through the self-help method.

The intervention "Everything under control" is available in a booklet for those patients who do not have access to the Internet or who prefer the booklet over the web based therapy. The content of the booklet is similar to the web based intervention. Coaching will be via telephone or e-mail.

The intervention "Everything under control" is offered to all patients in the experimental treatment arm: most patients are expected to suffer from psychological distress during the acute phase of the transplant [8,24]. The self-help intervention is offered as pro-active support in coping with this psychological distress.

#### Diagnostic evaluation, standardized interview, and problem analysis

After completing the Internet-based self-help program, all patients are screened for psychological distress. In order to cover the entire range of mood states associated with psychological distress, three instruments will be used measuring symptoms of psychological distress. These instruments are the Patient Health Questionnaire (PHQ-9), the Hospital Anxiety and Depression Scale (HADS), and the State-Trait Anxiety Scale: state version (STAI-state). For details on the measurement instruments, see section 'Assessment' below.

Psychological distress is defined as a score ≥10 on the PHQ-9, or ≥8 on the HADS (anxiety), or ≥8 on the HADS (depression), or ≥40 on the STAI (state) (adapted from Lee [8]).

#### Step 2: Contracting, individual face-to-face counseling, medication, or referral to other services

Patients meeting the criterion for psychological distress after completion of the Internet-based self-help program are offered further treatment. It is expected that approximately 50% of the patients will meet this criterion [8].

A collaborative team consisting of consultant psychiatrist, consultant psychiatric nurse, nurse practitioner (department of hematology), hematologist and patient is formed. The team evaluates the patient's need for treatment and develops the treatment plan. The nurse practitioner coordinates the team efforts, the development of the treatment plan and the execution of it. The team meets to develop tentative treatment options and to evaluate outcome of treatment.

Diagnostic evaluation is made by the consultant psychiatrist. The anxiety and depression modules of the Composite International Diagnostic Interview (CIDI) are administered for the classification of symptoms [25]. Additionally a standardized interview is held by the consultant psychiatric nurse, assessing the impact of distress on quality of life and the need for treatment. For this purpose, the Camberwell Assessment of Need (CAN) is used, measuring met and unmet needs on 22 domains of living [26]. The nurse practitioner and the consultant psychiatric nurse score case complexity and resulting care needs, using the Intermed [27,28]. Results of the diagnostic evaluation, standardized interview and scoring of case complexity are used by the collaborative team to analyze the existent problems, to evaluate the need for treatment and to develop tentative treatment options. The diagnostic evaluation, standardized interview and problem analysis are performed in a maximum of 4 weeks.

When treatment options have been developed, the patient is invited for an appointment with the consultant psychiatric nurse. In this meeting, the results of the problem identification and analysis are presented, personalized goals are identified and the patient is offered a choice between several treatment options (see below). The patient and consultant psychiatric nurse decide on the treatment, which matches the patient's problems, needs and preferences (contracting). The shared decision is written down in the personalized treatment plan: the treatment plan specifies identified problems, need for care, personalized goals, tailored treatment, and times for evaluation. The patient receives a copy of the personalized treatment plan and is invited to use the plan in staying focused on the personalized goals.

The following treatment options are available:

##### A) Individual face-to-face counseling

Face-to-face counseling is provided by the consultant psychiatric nurse, with the individual treatment plan serving as a guide. The treatment consists of problem solving treatment (see above), with a maximum of six sessions. The consultant psychiatric nurses have been thoroughly trained in problem solving treatment, and will follow a manual in delivering treatment [29,30]. If indicated, the partner will be involved in counseling. This decision will be made by the collaborative team.

##### B) Medication

Medication is prescribed by the consultant psychiatrist as needed. Suggested medication includes, among others, SSRI's and benzodiazepines. Occasionally, antipsychotics or mood stabilizers may be needed.

##### C) Referral

If an indication exists, the team refers the patient to health care or social services, e.g. physiotherapy, social work, or psychotherapy.

Step 2 including contracting and implementation of treatment options lasts for a maximum of 10 weeks.

#### II. Protocol care as usual

Care as usual consists of the following elements: If the patient brings up any problem, the hematologist interviews the patient (on an ad hoc basis, no formal screening for distress). During regular visits to the department of Hematology, hematologists and nurses provide emotional support and advise patients on how to cope with impairments of quality of life, on an ad hoc basis. If urgent problems emerge, the patient is referred to other services.

#### III. Contrast between interventions

A marked contrast between stepped care and care as usual exists. Key elements of the contrast include:

- Internet-based self-help program, based on the principles of problem solving therapy *versus *no self-help program.

- Formal screening for psychological distress *versus *ad hoc interview if a patient brings up any problems.

- Collaborative team (consultant psychiatrist, consultant psychiatric nurse, nurse practitioner, hematologist and patient) coordinated by nurse practitioner *versus *ad hoc care delivered by hematologist and nurse.

- Diagnostic evaluation (consultant psychiatrist), standardized interview assessing psychological distress (consultant psychiatric nurse) and problem analysis *versus *non-standardized interview performed by hematologist and nurse.

- Contracting *versus *no contracting.

- Individual face-to-face counseling, medication, or planned referral to other services *versus *support, advice, and referral to other services on ad hoc basis.

### Assessment

Assessments are made at baseline, 13 weeks, 30 weeks and 42 weeks post transplant.

#### Sociodemographic data

The following sociodemographic data are collected: age, gender, social status, employment, and Dutch vs. non-Dutch origin.

#### Medical-somatic data

Collected medical-somatic data are: diagnosis, time from diagnosis to auto-SCT, cancer treatment (pre-treatment, relapse vs. first line treatment), proceeding to allogeneic SCT within six months after auto-SCT (only in multiple myeloma group), hematological recovery data, number of platelet transfusions and packed cell transfusions, and survival/relapsed disease with concomitant second/third line treatment/death.

#### Main outcome variables

Psychological distress is measured by the Hospital Anxiety and Depression Scale (HADS). The HADS consists of 14 questions. There are two subscales assessing anxiety and depression, respectively. The two scales can be combined into one scale assessing psychological distress. The HADS does not contain items which might also be symptoms of physical illness (such as loss of appetite) [31-33]. This instrument has shown to be reliable, valid, and responsive, and has been widely used in research on cancer patients (e.g. [24]).

Quality of life (primary outcome: physical role function) is measured by the European Organisation for Research and Treatment of Cancer - Quality of Life Questionnaire - C30 (EORTC-QLQ-C30) version 3.0. The EORTC Quality of Life Questionnaire is an integrated system for assessing the health-related quality of life of cancer patients. The core questionnaire incorporates five functional scales (physical, role, cognitive, emotional, and social), three symptom scales (fatigue, pain, and nausea and vomiting), a global health status and quality of life scale, and a number of single items assessing additional symptoms commonly reported by cancer patients (dyspnea, loss of appetite, insomnia, constipation and diarrhea) and perceived financial impact of the disease. The instrument has been shown to be valid, reliable, and responsive to change [34].

#### Secondary outcomes

As secondary outcome variables, depression and anxiety are assessed by the Patient Health Questionnaire (PHQ-9) (depression) [35] and the State-Trait Anxiety Scale: state version (STAI-state) (anxiety) [36]. To measure health-related quality of life, the Medical Outcomes Study (MOS) 36-item Short Form Survey (SF-36) is used [37]. Other questionnaires are the Dutch General Self-efficacy Scale (DGSS) for measuring the belief of patients in their ability to function independently [38], the Social Problem Solving Skills-Revised (SPSI-R) to assess problem solving skills [39], and the Social Support List to measure the interactions and discrepancies that people experience in receiving social support from their direct environment [25]. At 42 weeks, patients' evaluation of care is assessed with the 'GGZ-thermometer', i.e. a Dutch instrument to measure patients' satisfaction with provided health care and with the quality of health care services [40]. The validity and reliability of these instruments have been described elsewhere [35,36,38-44].

#### Diagnostic evaluation and problem analysis

The evaluation of the patient's need for treatment prior to step 2 is made with the following instruments. Diagnostic evaluation is established with the anxiety and depression modules of the Composite International Diagnostic Interview (CIDI) [25]. The CIDI classifies diagnoses according to DSM-IV criteria [45]. For the assessment of care needs, two instruments are used: the Intermed [27,28] for assessing case complexity and resulting care needs, and the Camberwell Assessment of Need (CAN) [26] for detecting (un)fulfilled health and social needs in people with mental illness.

#### Process of care

A checklist is used to collect data on process of care: number of visits to an outpatient clinic, number of (re-)admissions, length of stay, second/third line treatment, content of stepped care (step 1 and step 2), and co-interventions (somatic and psychological treatment). This checklist was developed specifically for the trial.

### Statistical analysis

Primary outcomes are psychological distress as measured with the HADS and physical role function as measured with the EORTC-QLQ-C30. These primary outcomes reflect the central hypothesis of the study: stepped care is hypothesized to result in improvement in psychological distress, and thereby in improvement of functional status (see figure 1). Secondary outcome measures include: other measures of psychological distress; other aspects of quality of life; cognitions and coping.

Data are analyzed according to the intention to treat-principle. In a secondary analysis, a per protocol analysis will be performed. Baseline comparability of the experimental and control group is evaluated with descriptive statistics. The difference in outcome between stepped care and care as usual is evaluated by means of (mixed model) analysis of covariance. The randomization strata (hospital and diagnosis) will be included as covariates. In addition, a group*time-interaction term will be entered into the model to test for a difference in treatment effect over time. In analyzing a specific outcome variable, the baseline score of that variable is used as covariate.

If shown to be effective, we will explore whether patient characteristics (diagnosis, psychological distress, aspects of quality of life, cognition, coping, social support, and case complexity) moderate outcome of stepped care, using analyses of interaction between patient characteristics and treatment (stepped care vs. care as usual). Furthermore, we will explore which changes mediate outcome, by analyzing whether (a) change in psychological distress, (b) change in cognition, or (c) characteristics of the process of care mediate the outcome of stepped care, using the Baron and Kenny approach towards mediation and the Sobel test.

Both the analysis of moderating factors and the analysis of mediating factors is explorative in nature. This study is powered to answer the primary research question, i.e. the outcome of stepped care for psychological distress on functional status.

### Sample size

The power calculation concerns the comparison at T30 compared to T0 between the two groups (stepped care vs. care as usual). A recent meta-analysis on problem solving therapy for mental and physical health problems has documented an effect size of d = 0.54, compared to treatment as usual (compared to waiting list or no treatment, the effect size was d = 1.37; compared to attention placebo, the effect size was d = 0.54). Setting d = 0.5, alpha = 0.05 (two tail), beta = 0.80, the required sample size is 2 × 64 = 128 patients

A second power calculation was made concerning the difference in treatment effect over time (T0, T13, T30, and T42). Setting the within-subject correlation coefficient (rho) at 0.5, the required sample size following from this calculation is 2 × 42 = 84 patients.

The exclusion rate is estimated at 30% (primarily due to no informed consent). A detailed analysis of historical data has shown that the loss due to inclusion in other studies and mortality is 20%. The drop out rate is estimated at 20%. Consequently, 286 patients need to be invited for the study. On an annual basis, 80 autologous transplants are expected. Therefore, the inclusion period is 43 months.

## Discussion

This study design has several innovative characteristics. As to our knowledge, this is the first study in which the outcome of an intervention for psychological distress in patients with hematological malignancy treated with auto-SCT is evaluated in a randomized controlled protocol. Auto-SCT following high dose chemotherapy is a stressful treatment, leading to high levels of psychological distress [1]. Psychological follow up for patients treated with auto-SCT is in general not being systematically pursued; emotional support, advising on coping problems, and referring to other services usually occur on an ad hoc basis. To improve the quality of care and in order to reach more patients suffering from psychological distress following intensive hematological cancer treatment, it is essential to systematize and extend the follow up care.

The intervention in our study has been developed to improve patients' self-management and has been tailored to the needs of our specific patient group. Self-management is intended to help patients adjust to their condition and to improve their quality of life. The aims are to give patients control over their life and to obtain a proactive attitude in the patient [16]. Patients who are treated with auto-SCT for hematological malignancy, could therefore highly profit from improved self-management.

Furthermore, since psychological distress is a predictor of functional status in patients with hematological malignancy treated with auto-SCT [2-5], it is expected that the intervention will result in improvement of functional status if the intervention is successful in reducing psychological distress. The study is powered to evaluate the effect of the intervention on both psychological distress and functional status. Because (immuno-)chemotherapy and SCT have a strong impact on functional status, improved functional status as a consequence of reduced psychological distress would be a highly desirable outcome.

A third innovative characteristic of the present study is that the intervention aims at efficiency both in efforts and costs. This is reflected in the choice for a stepped care program, in which patients start with the least intensive treatment that is most likely to work, with more intensive and costly interventions reserved for those insufficiently helped by the initial self-help intervention. It is expected that most patients will be sufficiently helped by the Internet-based treatment, offered in step 1 [8]. Since there is minimal therapist contact, the therapist-time is being extremely reduced, the costs are low and there is no need for a waiting list [46]. Patients can systematically work on their self-management skills at any place and time they prefer.

Some patients will need more help than the Internet-based self-help program can offer them and will therefore enter step 2. A strength of this stage of the program is the multidisciplinary approach with a clear distribution of tasks and the nurse accomplishing a coordinating role. Multiple professional disciplines are brought together to treat the patients in the aftermath of disease. The collaborative team will evaluate the patients' need for treatment from various perspectives and subsequently develop the treatment plan. This collaboration of the various disciplines is coordinated by a nurse practitioner. Consequently, the professionals have the opportunity to deliver care efficacious and efficiently, and patients are assured of tailored care.

In the implementation of the stepped care program, nurses take up a central role. Especially during the phase of diagnostic evaluation and problem analysis, and during step 2, the nurse's contribution to treatment and care is essential. The nurse not only coordinates the collaborative team efforts, the development of the treatment plan, and the execution of it, but also assesses patients' case complexity and resulting care needs. Furthermore, the problem solving treatment sessions during step 2 are provided by a consultant psychiatric nurse.

The study also has some limitations that have to be taken into account. It could be that the most vulnerable patients will drop out of the experimental group because of difficulties completing the intervention program. This could reflect selective drop-out and lead to differences between the experimental and control group, because participants in the control group do not have to adhere to an intervention program, but only have to complete questionnaires. The data will be analyzed according to the intention to treat-principle. As a result, we will estimate the effects of allocating an intervention in practice, and not the effects in the subgroup of participants who adhere to the program. During the study, the drop out and (non-)compliance will be monitored. In a secondary analysis, patients completing treatment will be analyzed (per protocol analysis): this will allow us to estimate the effect of the intervention as such. Another limitation could be the length of the recruitment period. Given the long recruitment period, changes in medical treatment cannot be excluded. This may have impact on the intervention, although it is expected that patients in the intervention group and the control group will be affected in the same way.

If our trial shows a successful outcome, the intervention will be available for use in clinical practice. Results of this study will be available in 2014.

## Ethical considerations

The study protocol has been approved by the Medical Ethical Committee of VU University Medical Center, Amsterdam, the Netherlands. All patients gave written informed consent.

## Competing interests

The authors declare that they have no competing interests.

## Authors' contributions

JD, BM, OV, ATFB, PC, AB, PO, CE and AMJB contributed to the design of the study. The study is being coordinated by JD and BM. The present manuscript was drafted by AMJB, BM, and JD. All authors contributed to critical revision of the manuscript for important intellectual content. All authors read and approved the final manuscript.

## Pre-publication history

The pre-publication history for this paper can be accessed here:

http://www.biomedcentral.com/1471-2407/10/361/prepub
